# TORC2 mediates the heat stress response in *Drosophila* by promoting the formation of stress granules

**DOI:** 10.1242/jcs.168724

**Published:** 2015-07-15

**Authors:** Irena Jevtov, Margarita Zacharogianni, Marinke M. van Oorschot, Guus van Zadelhoff, Angelica Aguilera-Gomez, Igor Vuillez, Ineke Braakman, Ernst Hafen, Hugo Stocker, Catherine Rabouille

**Affiliations:** 1 Institute of Molecular Systems Biology, ETH Zurich, Zurich 8093, Switzerland; 2 Hubrecht Institute of the KNAW and UMC Utrecht, Uppsalalaan 8, Utrecht 3584 CT, Netherlands; 3 Cellular Protein Chemistry, Utrecht University, Padualaan 8, Utrecht 3584 CH, The Netherlands; 4 Department of Cell Biology, UMC Utrecht, Heidelberglaan 100, Utrecht 3584 CX, The Netherlands

**Keywords:** *Drosophila* S2 cells, TORC2, Rictor, Sin1, Heat stress, Akt, PKB, Heat-shock protein, SAPK, Stress granules, Translation

## Abstract

The kinase TOR is found in two complexes, TORC1, which is involved in growth control, and TORC2, whose roles are less well defined. Here, we asked whether TORC2 has a role in sustaining cellular stress. We show that TORC2 inhibition in *Drosophila melanogaster* leads to a reduced tolerance to heat stress, whereas sensitivity to other stresses is not affected. Accordingly, we show that upon heat stress, both in the animal and *Drosophila* cultured S2 cells, TORC2 is activated and is required for maintaining the level of its known target, Akt1 (also known as PKB). We show that the phosphorylation of the stress-activated protein kinases is not modulated by TORC2 nor is the heat-induced upregulation of heat-shock proteins. Instead, we show, both *in vivo* and in cultured cells, that TORC2 is required for the assembly of heat-induced cytoprotective ribonucleoprotein particles, the pro-survival stress granules. These granules are formed in response to protein translation inhibition imposed by heat stress that appears to be less efficient in the absence of TORC2 function. We propose that TORC2 mediates heat resistance in *Drosophila* by promoting the cell autonomous formation of stress granules.

## Abstract (precis)

**Highlighted Article:** The *Drosophila* TOR complex 2 is necessary for the formation of heat-induced cytoprotective stress granules, which can explain the heat sensitivity of TORC2 mutant flies.

## INTRODUCTION

Target of rapamycin (TOR) is a conserved serine/threonine kinase of the phosphoinositide 3-kinase (PI3K)-related kinase family, and functions in two distinct complexes, TOR complex 1 (TORC1) and TOR complex 2 (TORC2). Each complex comprises the kinase along with specific regulatory subunits that give the kinase its functional specificity and structural distinction. The core adaptor proteins of TORC1 are Raptor and LST8, whereas LST8, Rictor and Sin1 are the conserved components of TORC2. Removing either of the proteins from a cell destabilizes the TORC2 complex and inhibits its kinase activity (Frias et al., 2006; Jacinto et al., 2006, 2004; Kim et al., 2002; Loewith et al., 2002; Sarbassov et al., 2004).

Since its original discovery in screens for rapamycin suppressors (Heitman et al., 1991; Sabatini et al., 1994), TOR has been extensively studied in the context of TORC1, and has been shown to stimulate key anabolic cellular processes and inhibit the degradative pathway of autophagy (reviewed in Dibble and Manning, 2013; Loewith and Hall, 2011; Soulard et al., 2009) with crucial roles in metabolic diseases, cancer and aging (Cornu et al., 2014; Sabatini, 2006; Zoncu et al., 2011). TORC1 is widely regarded as the central node in cell growth control; its activity is dependent on growth factors and nutrient availability, and it is generally shut down in times of stress (Li et al., 2010; Reiling and Sabatini, 2006; Sancak et al., 2010; Sengupta et al., 2010; Urban et al., 2007).

Unlike TORC1, TORC2 is less well understood and knowledge on upstream cues regulating its activity is scarce. Its role in growth under normal conditions is minor (Hietakangas and Cohen, 2007; Soukas et al., 2009; Wang et al., 2012). In lower eukaryotes, TORC2 is activated upon nitrogen starvation, osmotic, heat and oxidative stress and DNA damage (Ikeda et al., 2008; Schonbrun et al., 2009; Weisman and Choder, 2001), and the TORC2 response to these environmental stresses is related to its likely ancient role in cellular signalling (Oh and Jacinto, 2011). TORC2 also has a role in actin cytoskeleton rearrangements (Schmidt et al., 1996) through PKCα, and RhoA- and Rac1-mediated pathways (Jacinto et al., 2004; Sarbassov et al., 2004). Recently, it has also been implicated in gluconeogenesis and sphingolipid metabolism, as well as apoptosis (Betz and Hall, 2013). The Akt (also known as PKB) family of protein kinases (Akt1 in *Drosophila*, hereafter referred to as Akt; there are three isoforms in mammals) are membrane-associated kinases from the family of AGC kinases, with well described roles in cell growth, metabolism and stress (Manning and Cantley, 2007; Scheid and Woodgett, 2001), and are one of the best characterized downstream targets of TORC2. *In vitro*, TORC2 has been shown to directly phosphorylate the hydrophobic loop of Akt1 (S473 in mammalian AKT1 or S505 in *Drosophila*), thereby increasing its kinase activity (Sarbassov et al., 2005).

There are three well-studied stress response mechanisms in cells. The first is mediated by the stress-activated protein kinases (SAPKs), the p38, JNK and Erk family kinases, either to protect the cell or to prime it for apoptosis (Johnson and Lapadat, 2002; Nadeau and Landry, 2007). The second response is the rapid upregulation of transcription of genes encoding heat-shock proteins (HSPs), which act as chaperones for cellular proteins to protect them against misfolding and aggregation in stressful conditions (Lindquist, 1986). The third mechanism includes responses that regulate translation and mRNA turnover. It is well established that heat exposure, oxidative stress and starvation induce the attenuation of bulk protein translation, polysome disassembly and accumulation of untranslated mRNAs. These are stored in cytoplasmic ribonucleoprotein particles (RNP) known as stress granules (Kedersha et al., 1999) along with translation initiation factors and RNA-binding proteins. From stress granules, stalled mRNAs can also be transported to the P-bodies (a different type of RNP that contains RNA decay machinery) for degradation, or upon stress relief, transferred back to polysomes for translation re-initiation (Anderson and Kedersha, 2008). Besides serving as transient protective storage of translation initiation components, the stress granules have also been suggested to serve as a transient station for the SAPKs and other pro-apoptotic kinases under stress, which is regarded to be a protective cellular mechanism against apoptosis (Arimoto et al., 2008; Thedieck et al., 2013). Whether TORC2 acts on these pathways in stress is not known.

Here, we show that TORC2 is specifically required for heat resistance *in vivo* as *Drosophila melanogaster* mutants for TORC2 components are selectively sensitive to heat stress. This sensitivity is accompanied by the reduced phosphorylation of Akt mirrored by the loss of the protein itself. By contrast, Akt phosphorylation is enhanced by heat in wild-type *Drosophila* larvae and cultured cells, showing that TORC2 is activated. Whereas the stress kinase and the HSP branches of the stress response are not affected, we show that the heat-induced stress granule formation is significantly delayed upon loss of TORC2 function, both in cells and in animals, and that a reduction of translation inhibition imposed by heat stress might be a cause for this delay. Taken together, we propose that under heat stress conditions, TORC2 promotes survival by enabling stress granule assembly.

## RESULTS

### Generation of a *Drosophila Rictor* mutant

**Fig. 1.**
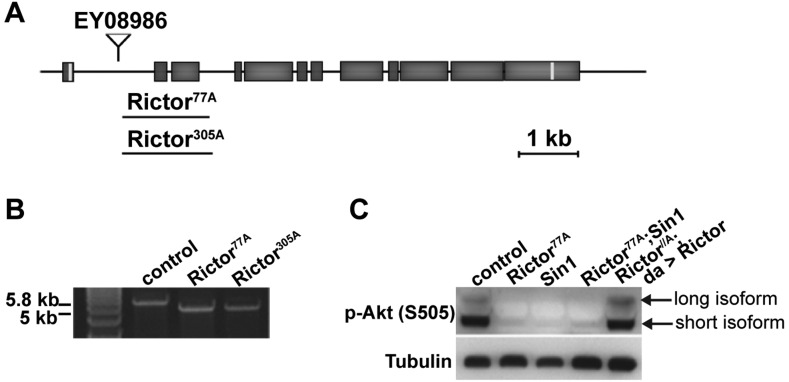
***Rictor* and *Sin1* mutant alleles.** (A) Schematic representation of the *Rictor* locus and the mutant alleles *Rictor^77A^* and *Rictor^305A^*. (B) PCR product of the *Rictor* open reading frame (ORF) amplified from cDNA of *Rictor* mutant and control flies. In the deletion mutants, the length of the *Rictor* ORF is 757 bp shorter than in the control, resulting in a premature stop codon after 58 amino acids. (C) Western blot visualization of Akt phosphorylation (p-Akt) on S505 in lines 1A (control), the *Rictor^77A^* mutant, the *Sin1* mutant, the double *Rictor^77A^*; *Sin1* mutant and in the *Rictor^77A^* carrying a rescuing a *Rictor* transgene (*da>Rictor*). Note that p-Akt is reduced in *Rictor* and *Sin1* single mutants and in *Rictor*; *Sin1* double mutants. Reduced phosphorylation is rescued with ubiquitous expression of *Rictor*.

To study the role of TORC2 in *Drosophila*, we generated *Rictor* mutant flies by mobilizing the EP-element EY08986 located in the first intron of the *Rictor* locus (CG8002) and screened for imprecise excisions. We obtained two independent deletions, *Rictor^77A^* and *Rictor^305A^*, which were of similar size (1.4 kb) and removed exons 2 and 3 (Fig. 1A). The *Rictor* mRNA produced by both mutations is 757 nucleotides shorter and generates a premature stop codon after 58 amino acids (Fig. 1B). A precise excision allele recovered in the screen was used as control throughout this study (control^1A^). As previously observed (Hietakangas and Cohen, 2007), loss of *Rictor* function in homoallelic and heteroallelic combinations as well as in hemizygous males resulted in viable flies with no obvious morphological defects, but that were slightly reduced in size.

As demonstrated in mammalian cells (Sarbassov et al., 2004), phosphorylation on S505 in the hydrophobic motif of Akt was compromised in *Rictor* mutants and was restored by ubiquitous expression of *Rictor* (Fig. 1C, right-most lane). Likewise, S505 phosphorylation was nearly absent in *Sin1* mutants, another specific component of TORC2 (Hietakangas and Cohen, 2007; Yang et al., 2006) (Fig. 1C). Taken together, this shows that the mutant we generated is a classical TORC2 mutant.

### *Rictor* and *Sin1* homozygous mutants are heat sensitive

**Fig. 2.**
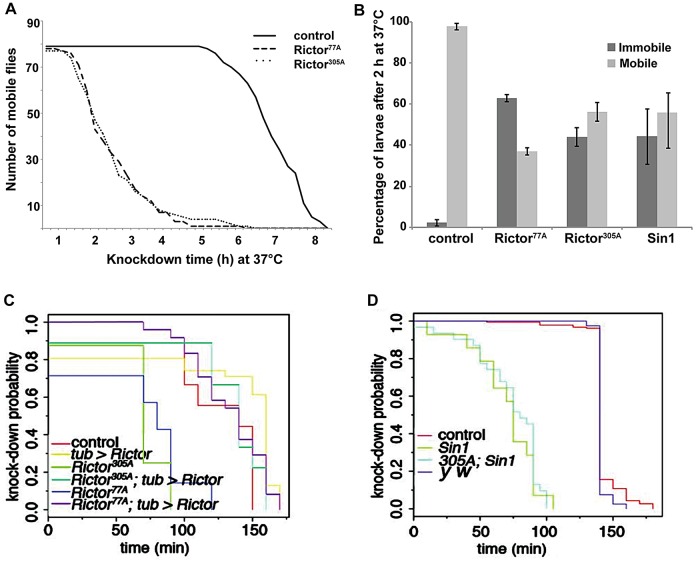
**TORC2 mutant flies and larvae are sensitive to heat stress.** (A) Mobility curve of control (1A) and *Rictor* mutants (77A and 305A) adult flies exposed to 37°C, expressed as number of mobile flies versus time elapsed. Note that both *Rictor* mutants behave similarly and that they are different from the control line (*n*=80 for each genetic background). (B) Control (1A, *n*=79), *Rictor* (77A, *n*=83; 305A, *n*=74) and *Sin1* (*n*=103) mutant larvae were exposed to 37°C for 2 h and the number of mobile versus immobile (paralyzed) larvae was counted. The graph shows the mean±s.d. of three independent experiments. (C) Quantification of the sensitivity to heat stress of control flies (1A, *n*=117, red curve), *Rictor^77A^* (*n*=131, blue curve) and *Rictor^305A^* (*n*=99, green curve) and of the rescue by the ubiquitous expression of *Rictor* (by means of *tub-Gal4* [*tub>Rictor* (*n*=31, yellow curve); *Rictor^77A^*; *tub>Rictor* (*n*=24, violet curve); *Rictor^305A^*; *tub>Rictor* (*n*=9, turquoise curve)]. *P*<0.01, *tub>Rictor* versus control; *P*<0.001, *Rictor^77A^; tub>Rictor* versus *Rictor^77A^*; *P*<0.001, *Rictor^305A^*; *tub>Rictor* versus *Rictor^305A^*. This is expressed as the probability of being paralyzed. Experiment is performed as in A but at 38.5°C. *P*-value for any mutant versus control is *P*<0.001. (D) Quantification of the sensitivity to heat stress of control flies (1A, *n*=186, red curve), *y w* (*n*=40, violet curve), *Sin1* mutant (*n*=14, green curve), and *Rictor^305A^*; *Sin1* double mutants (*n*=31, turquoise curve) as above. *P*-values for differences between *Sin1* and *Rictor*; *Sin1* mutants compared to the control are *P*<0.001.

The fact that flies lacking *Rictor* (and therefore TORC2 function) are viable is unexpected at first glance considering that in mice, *Rictor* and *Sin1* mutations are lethal (Jacinto et al., 2006; Shiota et al., 2006). However, TORC2 mutations that are lethal in *S. cerevisiae* are not lethal in *S. pombe* (Ho et al., 2005; Wilkinson et al., 1999). We set out to test whether the viability of *Rictor* mutant flies is modulated by challenging environmental conditions (heat stress, oxidative stress and starvation). We found that *Rictor* mutant flies are exclusively sensitive to heat stress (Fig. 2A,C) but not to oxidative stress or dry starvation (supplementary material Fig. S1A,B). Upon heat exposure, *Rictor* mutant adult males became sluggish and rapidly began to fall to the bottom of the vial (Fig. 2A,C). We also observed that, when immediately returned to normal temperature, they were able to recover, indicating that high temperatures caused paralysis rather than instant death. Similarly, *Rictor* and *Sin1* mutant larvae also display a similar heat sensitivity phenotype exemplified by their reduced or absent mobility as compared to control larvae of the same stage after exposure to 37°C (Fig. 2B). The heat sensitivity of the flies was rescued by ubiquitous *Rictor* expression (*tub-Gal4, UAS-Rictor*) (Fig. 2C). *Sin1* mutant adult flies, as well as the double mutant *Rictor*; *Sin1* animals (Fig. 2D) displayed the same sensitivity to heat, confirming that TORC2 function is required for the normal heat response.

### Akt phosphorylation on S505 and stability upon heat stress is TORC2 dependent

**Fig. 3.**
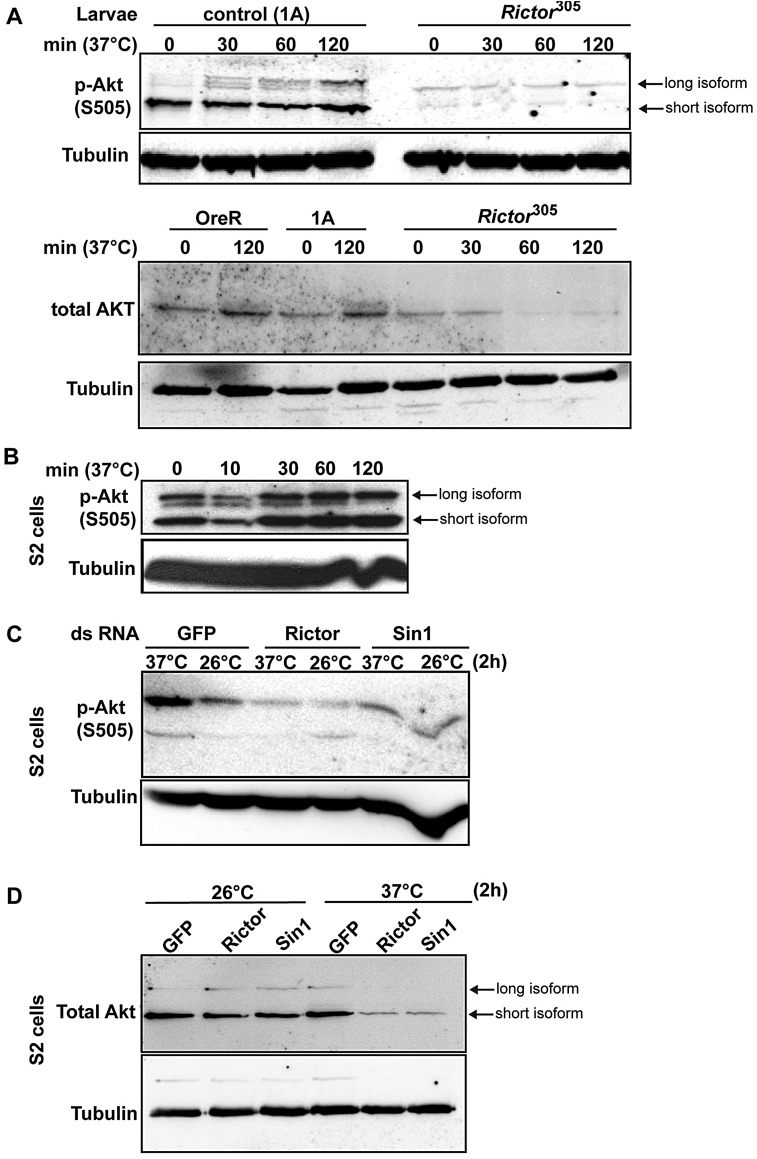
**Akt phosphorylation on S505 and stability upon heat stress are TORC2 dependent.** (A) Western blot of Akt phosphorylated on S505 (p-Akt)and total Akt in lysates of control and *Rictor^77A^* homozygous mutant larvae upon heat exposure for up to 2 h. Note that in *Rictor* mutants, the heat-induced Akt phosphorylation is not observed and that Akt is lost upon heat stress. (B) Western blot of p-Akt in lysates of S2 cells upon heat stress at 37°C for increasing time (up to 2 h). (C) Western blot of p-Akt in *GFP*-, *Rictor*- and *Sin1*-depleted S2 cells exposed at 37°C for 2 h. Note that p-Akt does not increase upon loss of TORC2 function. The lower band in this blot is non specific. (D) Western blot of total Akt in *GFP*-, *Rictor*- and *Sin1*-depleted S2 cells exposed to 26°C and 37°C for 2 h. Note that the Akt level is similar for all conditions at 26°C as well as for mock-depleted conditions at 37°C but dramatically drops in heat-exposed *Rictor*- and *Sin1*-depleted cells.

Given that TORC2 phosphorylates Akt (Fig. 1C) and that TORC2 mutants are heat sensitive, we assessed whether heat stress induces Akt phosphorylation. Of note, Akt protein exists in two isoforms in *Drosophila* but the role of the larger one has not been properly defined (Andjelkovic et al., 1995). Using extracts of control larvae exposed to 37°C for up to 2 h, we show that the phosphorylation of the two Akt isoforms increases with time without a change in its total amount (Fig. 3A).

We reproduced the heat-induced TORC2 activation in *Drosophila* tissue cultured S2 cells. There, the two Akt isoforms were also phosphorylated and the intensity of this phosphorylation increased upon heat treatment without a change in the total level of Akt (Fig. 3B), indicating a heat specific activation of TORC2. By contrast, *Rictor* mutant larvae (Fig. 3A) and S2 cells depleted of either *Rictor* or *Sin1* (Fig. 3C) did not show this increased Akt phosphorylation upon heat stress. Surprisingly, we found that this was paralleled by a strong reduction of the level of Akt protein, both in cells (Fig. 3D) and in larvae (Fig. 3A), possibly due to protein degradation. These results show that TORC2 is required to maintain Akt protein levels during heat stress, and re-enforce the link between TORC2 and heat stress.

### TORC2 does not mediate SAPK activation and the upregulation of HSPs

**Fig. 4.**
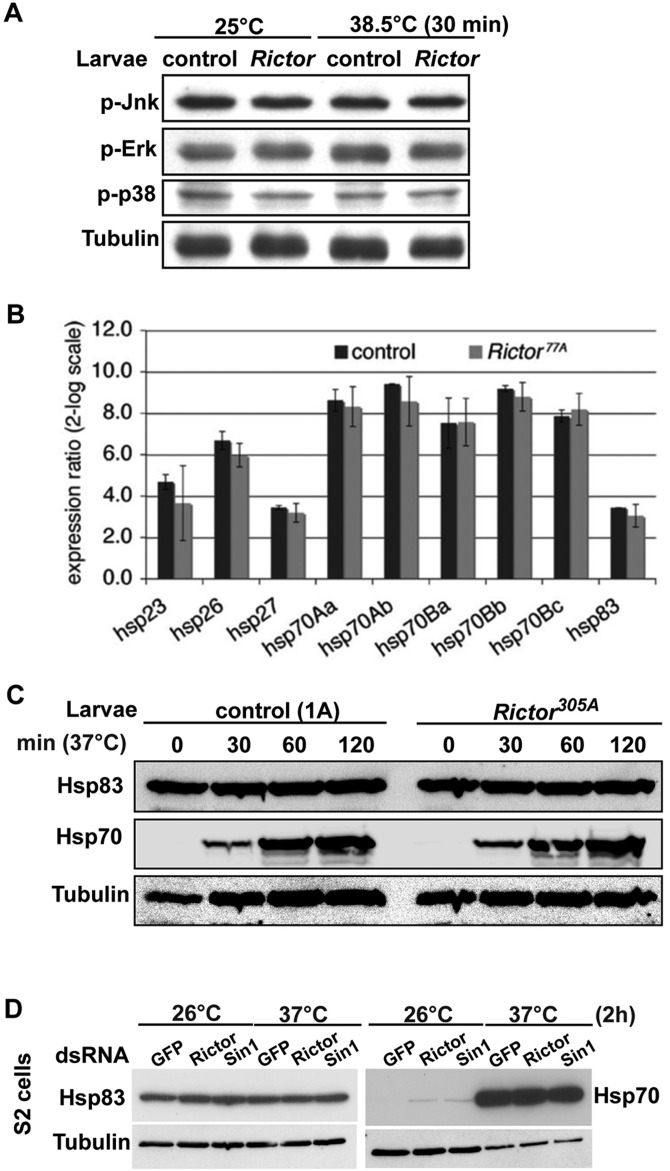
***HSP* gene expression and translation is not compromised in *Rictor* mutants.** (A) Western blot of phosphorylated p38 (p-p38), phosphorylated JNK (p-Jnk) and phosphorylated Erk1/2 (p-Erk) of control and TORC2 mutant larvae upon heat stress. Note that their phosphorylation does not change. (B) Quantitative RT-PCR of *HSP* gene expression upon heat treatment in control and *Rictor^77A^* mutant larvae. The data represent the average of two biological replicates from larvae raised and treated identically. The expression upon heat is represented relative to *HSP* expression levels under normal conditions. (C) Western blot of HSP83 and HSP70 in extracts of control (1A) and *Rictor^305A^* larvae exposed to 37°C for indicated time. (D) Western blot of HSP83 and HSP70 in lysates of mock-, *Rictor*- and *Sin1*-depleted S2 cells exposed to 37°C for the indicated time. Note that less lysate was loaded for HSP70 after exposure to 37°C to better appreciate possible differences.

A response to heat stress could be the TORC2-mediated phosphorylation of the major SAPKs [p38 (p38a–p38c), Erk1/2(Rolled) and Jnk (Basket)]. However, in our test conditions, this response does not change upon heat stress and, importantly, is not modulated by TORC2 (Fig. 4A). Therefore, we conclude that the SAPKs are not involved in the heat sensitivity downstream of TORC2. This suggests that the Akt fate in heat and upon TORC2 loss-of-function is specific and that the reported association of Sin1 with the SAPK pathway components (Oh and Jacinto, 2011) is independent of TORC2.

A second response to heat stress is the transcriptional activation and post-translational modifications of HSPs (Lindquist, 1986). We first checked whether HSPs are transcriptionally upregulated in response to heat in *Drosophila* larvae in a TORC2-dependent manner. Using quantitative real-time PCR (qRT-PCR), we show that the transcription of nine *HSPs* (including the five isoforms of *HSP70*) is activated by heat stress in wild-type larvae. However, the transcriptional upregulation of the tested *HSP* genes is unchanged in *Rictor* mutants (Fig. 4B), suggesting that the role of TORC2 in heat stress is not related to *HSP* transcription. We also tested the protein level of two HSPs. The protein level for the members of the HSP70 family was enhanced upon heat stress, as reported previously (Lindquist, 1986), and again TORC2 loss of function, whether in larvae or S2 cells, did not modify this (Fig. 4C,D). The HSP83 level did not change in wild-type larvae and S2 cells upon heat treatment and also remains unchanged in *Rictor* mutant larvae or *Rictor*- and *Sin1*-depleted cells (Fig. 4C,D). This confirms that the loss of TORC2 function does not modulate HSP83 and HSP70 levels.

Taken together, our results show that the heat-shock response of transcriptionally and translationally upregulating the HSP level is not modulated by TORC2. In fact, the HSPs HSP70 and HSP90 (the mammalian homolog of *Drosophila* HSP83) might even stabilize TORC2 during heat stress (Martín et al., 2008; Takai et al., 2010).

### TORC2 is specifically required for stress granule formation under heat stress in *Drosophila* cells and tissues

A third key response to heat stress is the attenuation of bulk protein synthesis (see Fig. 7, below), which leads to the accumulation of free untranslated mRNAs and their storage in stress granules (Anderson and Kedersha, 2008). The assembly of these granules has recently been observed upon heat stress in *Drosophila* both *in vivo* (Gareau et al., 2013; van der Laan et al., 2012) and in S2 cells (Farny et al., 2009).

**Fig. 5.**
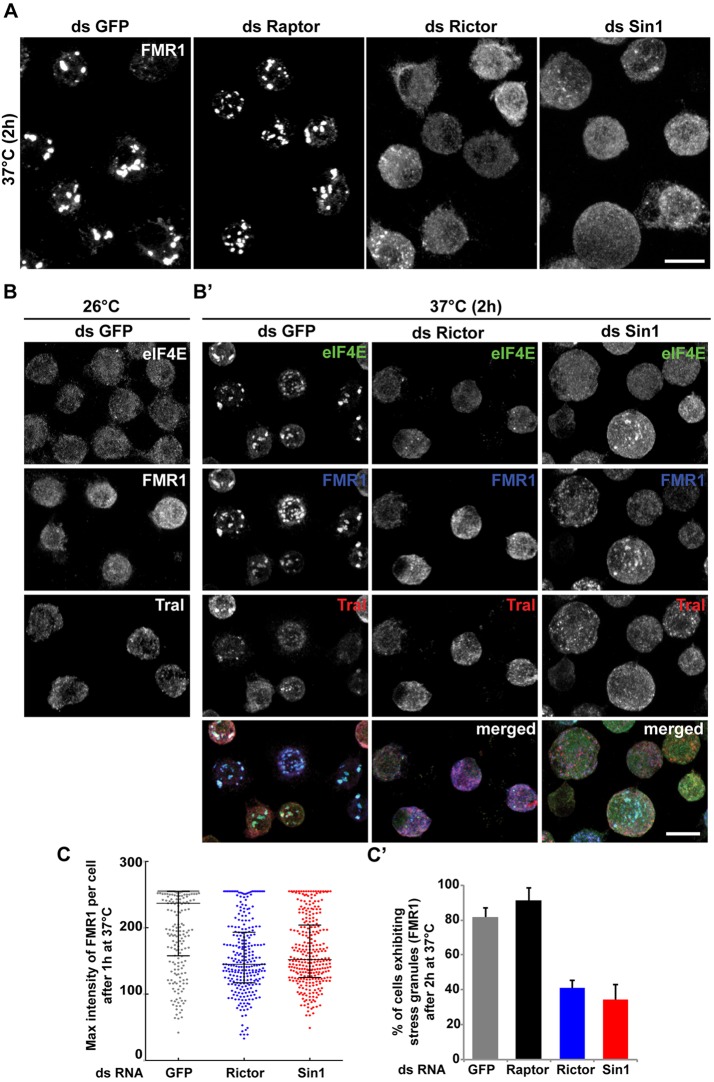
**Stress granule formation is delayed in TORC2 depleted S2 cells.** (A) Immunofluorescence visualization of endogenous FMR1 in S2 cells depleted for *GFP* (control, dsGFP), *Raptor* (TORC1), *Rictor* and *Sin1* (TORC2) and heat stressed at 37°C for 2 h. Note that in heat-stressed mock- and *Raptor*-depleted cells, FMR1 is found in stress granules, whereas in *Rictor-* and *Sin1*-depleted cells, FMR1 remains largely cytoplasmic. (B,B′) Immunofluorescence visualization of endogenous FMR1, eIF4E and Tral (P-bodies) in *GFP*-depleted S2 cells grown at 26°C (B) and in *GFP*-, *Rictor-* and *Sin1*-depleted S2 cells heat stressed at 37°C for 2 h (B′). Note that eIF4E colocalizes with FMR1 in stress granules upon heat stress and that Tral localization is not affected by heat stress and by depletion of the TORC2 components. Scale bars: 10 μm. (C­,C′) Quantification of stress granule formation in *GFP*-, *Raptor*-, *Rictor*- and *Sin1*-depleted S2 cells. The maximum FMR1 intensity per cell upon exposure at 37°C for 1 h (C) is represented. The error bar is the mean±interquartile range. The bar representation in C′ displays the mean±s.d. percentage of cells exhibiting stress granules in *GFP*-, *Raptor*-, *Rictor*- and *Sin1*-depleted cells upon exposure at 37°C for 2 h from three independent experiments. *P*<0.0005, *Rictor-*depleted versus *GFP*; *P*<0.005, *Sin1*-depleted versus *GFP*.

We therefore tested whether stress granule assembly in heat-stressed S2 cells is sensitive to loss of TORC2 components. Stress granule formation was monitored using two endogenous components. The RNA-binding protein Fragile mental retardation protein 1 (FMR1) (Gareau et al., 2013) (Fig. 5A) and the translation initiation factor eIF4E (Fig. 5B) were diffuse in the cytoplasm at 26°C (Fig. 5B) and colocalized in stress granules in S2 cells exposed to 37°C for 1 to 2 h (Fig. 5A,B′,C). Interestingly, P-bodies, a cytoplasmic assembly that mediates the basal RNA metabolism under basal growth conditions (see Introduction), marked here by the ‘like Sm’ (LSM) protein Tral, do not change upon heat stress (Fig. 5B′). They remain as small foci as previously described (Eulalio et al., 2007).

Upon depletion of *Rictor* and *Sin1*, stress granule assembly is inhibited, as assessed using both FMR1 and elF4E (Fig. 5A,B′). Short periods of heat exposure (between 1 and 2 h) drove the efficient assembly of stress granules in wild-type S2 cells, a response that was significantly reduced in both *Rictor*- and *Sin1*-depleted cells. Smaller structures were still occasionally visible but these were significantly reduced in number compared with the larger ones observed in mock-depleted cells (Fig. 5C,C′). However, this difference was no longer visible upon prolonged exposure to heat (3 h or more, not shown), suggesting that the loss of TORC2 induces a delay in stress granule assembly but does not prevent it completely. By contrast, the depletion of Raptor, the key component of the TORC1 pathway, does not alter stress granule formation (Fig. 5A,C). This suggests that one key response to heat, the formation of stress granules, is kinetically compromised upon loss of TORC2 but not TORC1 function.

**Fig. 6.**
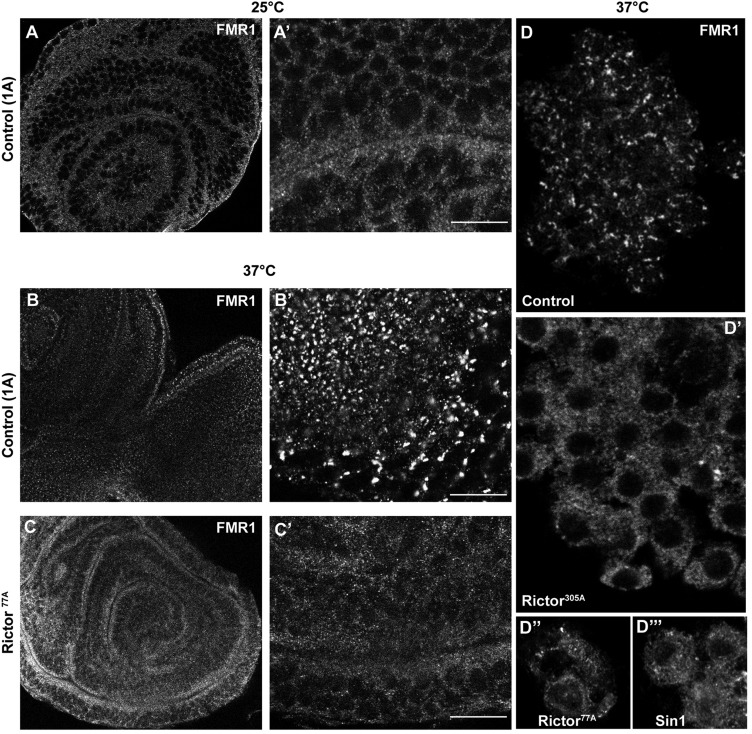
**Stress granule formation is impaired in *Drosophila* tissues.** (A–C′) Immunofluorescence visualization of FMR1 in imaginal discs of control larvae at 25°C (A,A′), larvae exposed to 37°C for 2 h (B,B′) and *Rictor^77A^* mutant larvae exposed at 37°C for 2 h (C,C′), in low (left) and high (right) magnification. (D) Immunofluorescence visualization of FMR1 in clusters of hemocytes from control (D), *Rictor^305A^* (D′), *Rictor^77A^* (D″) and *Sin1* (D‴) mutant third-instar larvae heat stressed for 2 h. Note that stress granule formation is reduced in mutants for TORC2 components. Scale bars: 10 μm.

To test whether stress granule formation could be the basis of the heat sensitivity observed in the animals, we monitored whether their formation in larval tissues exposed to heat is also TORC2 dependent. Control larvae exposed for 2 h at 37°C did exhibit stress granules in their tissues, especially in the imaginal discs where they were very prominent (Fig. 6A–B′). Strikingly, they did not form in discs dissected from *Rictor* mutant larvae upon the same conditions (Fig. 6C,C′), thus reproducing the pattern as observed in S2 cells. Similar to in imaginal discs, the hemocytes of heat-stressed control larvae also exhibited stress granules and their assembly was compromised in *Rictor* and *Sin1* mutant larvae (Fig. 6D–D‴). Interestingly, stress granules did not seemingly form in the fat body of control heat-stressed larvae. By contrast, heat stress induced stress granule formation in the brain of control larvae but this was not changed in TORC2 mutant animals (supplementary material Fig. S2). Although this suggests that stress granule formation and/or dynamics are not solely controlled by TORC2 components in different tissues, their impaired formation in certain tissues, such as in imaginal discs and hemocytes, could be the basis of the heat sensitivity of *Rictor* and *Sin1* mutant animals.

### TORC2 modulates the translation inhibition imposed by heat stress

As mentioned above, stress granules form in response to the accumulation of untranslated mRNAs due to the inhibition of protein translation imposed by stress. One possibility to explain the role of TORC2 in stress granule formation is that in the absence of its function, translation is not efficiently inhibited, thus maintaining the mRNAs engaged in the polysomes and lifting the need for mRNA storage.

**Fig. 7.**
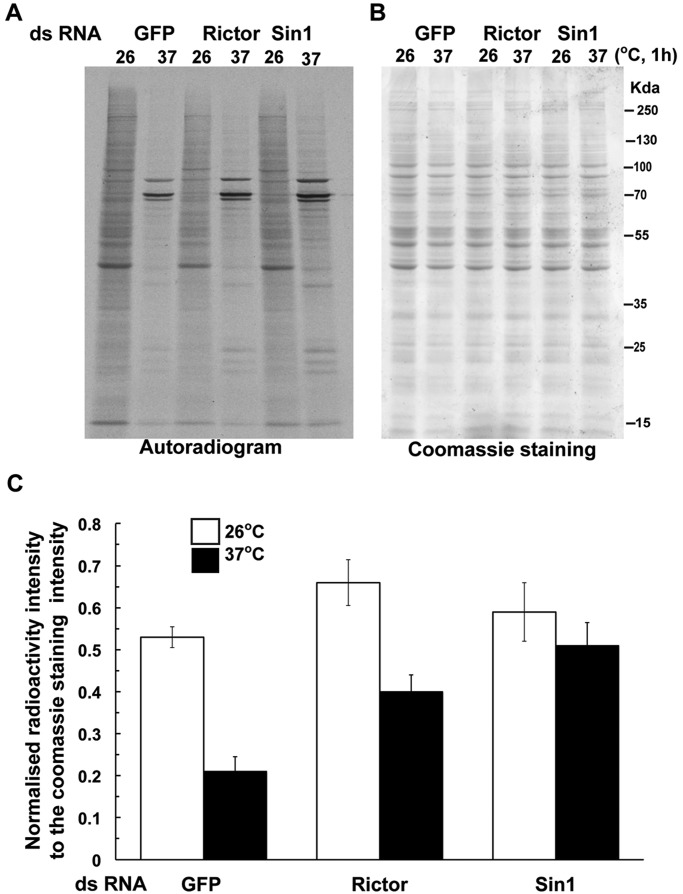
**Heat-induced protein translation inhibition is not impaired upon TORC2 loss of function.** (A) Autoradiogram of total proteins radiolabelled with a pulse of [^35^S]methionine for 15 min from lysates of mock (*GFP*)-, *Rictor*- and *Sin1*-depleted S2 cells after 55 min at 26°C and 37°C analysed using 10% SDS-PAGE. Note that the 26°C and 37°C patterns are similar for all cells. (B) Coomassie gel of fractionated proteins for the samples analysed in A. (C) Quantification of the autoradiography normalized to the Coomassie staining at 26°C and 37°C for mock-, *Rictor*- and *Sin1*-depleted S2 cells (as performed in A). Results are mean±s.d. (*n*=2).

To test this, we monitored protein translation in mock-, *Rictor*- and *Sin1*-depleted cells both at normal growth temperature (26°C) and after heat shock (37°C). As expected, protein translation was strongly inhibited (∼60%) after 1 h (Fig. 7A,B) and 2 h (data not shown) of exposure of mock-depleted cells to 37°C, except for two bands at ∼70–100 kDa that were strongly increased (presumably corresponding to HSPs) and a quadruplet of low molecular mass. Strikingly, the autoradiogram patterns were very similar in mock-, *Rictor*- and *Sin1*-depleted cells exposed to 37°C (Fig. 7A,B), showing that heat stress leads to the same change in translation pattern whether TORC2 function is present or not.

However, the overall protein translation appeared to be stronger in TORC2-depleted cells both in basal growing conditions and upon heat stress (Fig. 7C). This is especially true for the *Sin1* depletion, whereas *Rictor*-depleted cells were more similar to the mock-depleted ones. Overall, loss of TORC2 function leads to a more efficient translation. Whether, in the case of Rictor, this is sufficient to reduce the kinetics of the stress granule formation in TORC2 mutants, remains an open question.

### Stress granules have a cytoprotective function

Stress granules have been proposed to be cytoprotective during stress. When key factors required for their formation are depleted or mutated, cell survival during stress has been observed to be significantly lower, for instance Vgl during heat stress (Wen et al., 2010), FUS during hyperosmotic stress (Sama et al., 2013), importin α1 during arsenate stress (Fujimura et al., 2010) and 4E-BP1 during selenite poisoning (Fujimura et al., 2012). This is probably due to their role in preserving nascent mRNA from degradation as well as accumulating pro-apoptotic kinases to prevent to trigger apoptosis (Arimoto et al., 2008; Eisinger-Mathason et al., 2008; Kim et al., 2005; Kwon et al., 2007; McEwen et al., 2005). However, the mentioned experiments do not allow a clear distinction between the role of the factor itself from its role in stress granule formation.

**Fig. 8.**
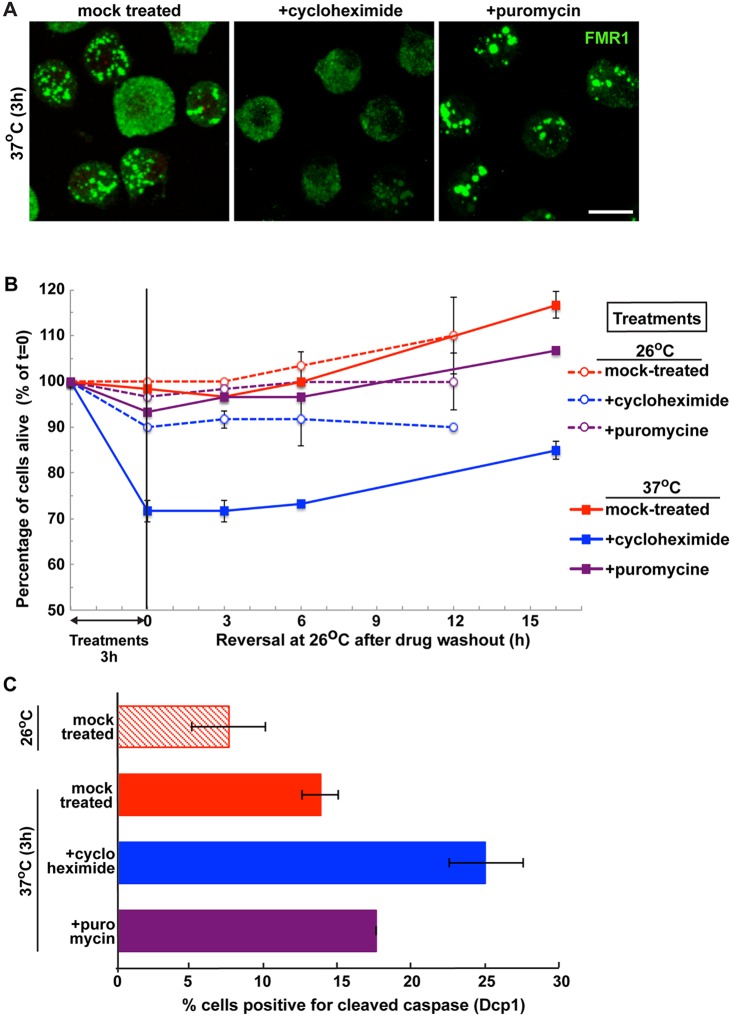
**Stress granule formation is a pro-survival mechanism.** (A) Immunofluorescence visualization of FMR1 in S2 cells incubated at 37°C for 3 h in the presence of cycloheximide and puromycine. Note that cycloheximide treatment completely blocks stress granule formation whereas puromycine does not. Scale bar: 10 µm. (B) Graph of cell survival (as percentage of cell that remain alive, mean±s.d., *n*=3) after 3 h incubation at 26°C or 37°C in the presence of drugs (treatment is as in A) followed by incubation at 26°C in the absence of drug. *P*-values between all the treatments (except for cycloheximide at 37°C) are above 0.13 making the difference insignificant. The *P*-value between cycloheximide at 37°C and the other treatments is below 0.0000001. (C) Percentage of cells positive for cleaved caspase 3 during treatment as in A. Results are mean±s.d. (*n*=3).

Here, we re-addressed the pro-survival role of stress granules upon heat stress under pharmacological conditions that prevent the formation of stress granules. We used cycloheximide, a drug that ‘locks’ ribosomes on mRNAs, thus preventing the formation of stress granules during stress (Fig. 8A) (Yamasaki et al., 2007). As cycloheximide inhibits protein synthesis, we also treated cells with puromycin, which leads to the dispersion of ribosomes away from mRNAs, thus allowing stress granule formation (Fig. 8A).

We found that mock- and puromycin-treated cells survived heat shock with hardly any cell death, whereas cycloheximide-treated cells had a significantly lower survival (Fig. 8B). This correlates very well with the number of pro-apoptotic cells (i.e. positive for cleaved caspase 3) (Fig. 8C), which is higher in the cycloheximide-treated cells than in the other two conditions. In the context of the molecular experiments mentioned above, these results support the notion that stress granules are pro-survival during heat stress. Hence, we speculate that stress granule formation prevents a heat stress sensitivity of the whole organism.

## DISCUSSION

Our results show that one key branch of the response to heat stress, the formation of stress granules, is delayed by the loss of TORC2 function both in *Drosophila* tissues and cultured cells. TORC2 is activated upon heat stress and mediates the formation of stress granules, likely required for heat resistance at the cellular level in *Drosophila*.

How TORC2 mediates stress granule formation is not clear. Heat stress is known to stimulate the inhibitory phosphorylation of the initiation factor eIF2α resulting in protein translation stalling (Farny et al., 2009 and M.Z. and C.R., unpublished results). However, this phosphorylation is not required for stress granule formation in *Drosophila* upon heat stress (Farny et al., 2009), so it is unlikely that TORC2 modulates this event.

Here, we show that stress granule formation is delayed by loss of TORC2 function and we suggest that this is due to a lift on the overall translation inhibition imposed by heat stress, and also under basal conditions. Depletion of TORC2 components appears to stimulate protein translation. This is in accordance with our observations that depletion of either *Rictor* or *Sin1* from *Drosophila* S2 cultured cells causes their increased proliferation (115%±10, mean±10, *n*=6) and cell diameter, respectively. This activation of translation upon loss of TORC2 function could be due to activation of TORC1, as observed previously in Kc cells, another *Drosophila* cultured cell line. There, depletion of *Rictor* elevates levels of the phosphorylated 4E-BP, a known target of TORC1 (Glatter et al., 2011).

Because *Rictor* and *Sin1* mutant flies are smaller in size than control animals (Hietakangas and Cohen, 2007 and I.J., E.H. and H.S., unpublished observations), it suggests that any translation activation potentially leading to an increase in cell growth and proliferation is tissue specific. This might mirror the tissue-specific response in stress granule formation that we report here. Such stimulated translation, even upon heat stress might delay or impair stress granule assembly. However, *Sin1* depletion has a much stronger effect on translation than *Rictor* depletion, yet stress granule assembly is inhibited to the same extent in both backgrounds. Hence, whether this lift in translation inhibition is the sole parameter impairing stress granule formation remains to be further investigated. In this regard, Rictor is found at the ribosomes interacting with RACK1 (Zinzalla et al., 2011), a selective mediator in stress granule function (Arimoto et al., 2008; Ohn et al., 2008). Thus, it remains to be determined whether TORC2 senses ribosomal activity and mediates the stress granule assembly on its own, rather than indirectly, by providing balance to TORC1.

Interestingly, the ribosomal localization of Rictor activates Akt, the TORC2 downstream kinase and we show here that Akt is activated upon heat stress both in animals and cell lines, in line with mammalian studies (Bang et al., 2000; Shaw et al., 1998). This heat-mediated activation is in line with the finding that *S. pombe* mutants for *Tor1* (the kinase of TORC2), *Sin1* and *Gad8* (encoding an Akt ortholog) are also sensitive to heat stress (Ikeda et al., 2008). This suggests that the TORC2–Akt signalling axis represents an ancient and conserved cellular mechanism to cope with heat stress.

Surprisingly, however, we find that TORC2 function not only modulates Akt phosphorylation but also its abundance. Strikingly, the absence of TORC2 function both in cells and larvae rapidly and significantly obliterates Akt, possibly through increased degradation. It is likely not due to a lower translation during stress, because translation is less inhibited in heat stress in the absence of TORC2 components. This correlates well with studies in mammalian cells, where PKCα, a second known downstream target of TORC2, and a small fraction of Akt are degraded by the proteasome ubiquitin pathway in cells depleted for TORC2 components. This is due to the lack of the phosphorylation by TORC2 that primes PKCα and Akt for ubiquitylation (Ikenoue et al., 2008; Oh et al., 2010). Whether and how Akt plays a role in stress granule formation in *Drosophila* remains to be investigated.

The TORC2-based mechanism we propose is different from the one described in mammalian cells (especially cancer cell lines) where TORC1 is a key player. Indeed, depletion of TORC1 components impairs stress granule assembly by reducing the phosphorylation of 4E-BP, subsequently preventing the formation of eIF4E–eIF4GI cap-dependent translational initiation complexes (Fournier et al., 2013). In S2 cells, however, we do not observe a direct role for TORC1 in stress granule formation. Neither *Raptor* depletion nor rapamycin treatment (data not shown) impairs stress granule formation upon heat stress. Whether this differential involvement of TORC1 and TORC2 in stress granule formation is cell- and tissue- dependent and acts through different pathways remains to be tested. Alternatively, the different mechanisms might suggest that mammals have evolved more sophisticated mechanisms to cope with stress. TORC1 has also been shown to be sequestered in stress granules during heat and other stresses (Takahara and Maeda, 2012; Thedieck et al., 2013; Wippich et al., 2013) where it suppresses its own apoptotic activity, corroborating its role in stress granule function.

The importance of studying environmental effects on signalling pathways, like the TOR pathway, is illustrated by the central role of these pathways in the progression of diseases, such as metabolic and neurological diseases or cancer (recently reviewed in Beauchamp and Platanias, 2013; Laplante and Sabatini, 2012). Elucidating the modulation of such pathways under different environmental conditions can potentially identify new targets and processes playing roles in the physiological or pathological regulation of cell survival.

## MATERIALS AND METHODS

### Fly strains

The *Rictor* deletion alleles were generated in a P-element excision screen using isogenized P(EPgy2)rictorEY08986 flies (Bloomington stock center, IN). A precise excision from the same screen was used as a control throughout this study. The *Sin1* allele was PBac(RB)Sin1e03756 (all from the Bloomington stock center). *tub-Gal4* or *da-Gal4* were combined with *UAS-Rictor* flies for the rescue experiments.

### Generation of the *UAS-Rictor* construct

*Rictor* was amplified from cDNA using the primers 5′-CACCATGGCCTCCCAACATTCCAG-3′ (Forward) and 5′-GGAGTTGTCACGCACAGTTGTC-3′ (Reverse), cloned into pENTR-D-TOPO (Invitrogen, Life Technologies, Zug, Switzerland), transferred into pTHW by Clonase reaction and injected into *y w* embryos.

### Stress tests on flies

Heat stress tests were conducted at 37°C or 38.5°C in a hybridization oven (Thermo Electron, ThermoFischer Scientific, Reinach, Switzerland). The flies were raised at 25°C in bottles under optimal crowding conditions. Males were separated from females upon eclosion, staged for 3 days and transferred into empty plastic vials (no more than 30 flies per vial). The vials were sealed with foam plugs soaked in water to mimic a humid environment. The knockdown was scored over time. Both males and females performed equally; data presented for *Rictor* and *Sin1* alleles are for males. For comparison to wild type, the *Rictor* precise excision allele was shown to perform similarly to the *y w* and Canton S strains. Oxidative stress (with 5% H_2_O_2_) and starvation tests were conducted as described previously (Jünger et al., 2003). Flies were collected as for heat stress tests.

### Statistical analysis of survival

In the stress resistance analysis in Fig. 2 and supplementary material Fig. S1, data were plotted as ‘number of flies alive at a certain time point’ and calculated using the statistical program R. Curves were estimated with the survfit function, which uses the Kaplan–Meier estimator. *P*-values were calculated with the log-rank test in R.

### Larval heat stress

A total of 15–25 third-instar 1A, *Rictor^305A^* and *Sin1* mutant larvae were placed in 3.5-cm plastic dishes filled up with fly food to keep the moisture optimal. The lid was fixed with tape so the larvae did not escape during the treatment. The dishes were placed at 25 or 37°C in an oven for increasing periods of time up to 2 h. The larvae were then assessed for their mobility. A mobile larva was a larva that crawls on its own or when nudged with a forcep (*n*=4). Note that at 25°C, all larvae were mobile. For immunofluorescence procedures, see below.

### S2 cells and heat stress

*Drosophila* S2 cells were cultured in Schneider's medium supplemented with 10% insect-tested fetal bovine serum (referred to as Schneider's) at 26°C as previously described (Kondylis and Rabouille, 2003; Kondylis et al., 2007). Heat stress was carried out by incubating the cells for 1–2 h at 37°C. Stress granule formation was assessed by immunofluorescence.

### RNAi depletion

A total of 0.7×10^6^ wild-type S2 cells were depleted by RNAi as previously described (Kondylis and Rabouille, 2003; Kondylis et al., 2007). dsRNAs for were generated with the Megascript T7 kit using the following sets of primers: *Raptor*: 5′-TTAATACGACTCACTATAGGGAGACGTATGGAACCGGAAGACAC-3′ and 5′-TTAATACGACTCACTATAGGGAGAGTATCCTCGGAGGTGGCAG-3′ or 5′-TTAATACGACTCACTATAGGGAGAGATCAAGAGGTGGCCTCCAG-3′ and 5′-TTAATACGACTCACTATAGGGAGAGCATGCCCAGGATCTGTATT-3′; *Rictor*, 5′-TTAATACGACTCACTATAGGGAGAACGAGTCCAACTCACAGGCT-3′ and 5′-TTAATACGACTCACTATAGGGAGAGTTGGCTGATGTCCGAAAAG-3′ or 5′-TTAATACGACTCACTATAGGGAGAACCGAGCGTAGTAGAGCAGC-3′ and 5′-TTAATACGACTCACTATAGGGAGAAGACAGAATCCAGCCGAGAA-3′; *Sin1*, 5′-TTAATACGACTCACTATAGGGAGAGATATGCTGGAAGCACCCAT-3′ and 5′-TTAATACGACTCACTATAGGGAGATCAGCTTGTTGTGGATCTGC-3′.

*Raptor* depletion was evidenced by its strong phenotype on cell growth and size (cell proliferation was down to 40% of mock depleted and the cells were 10% smaller in diameter). Conversely, *Rictor* depletion had a negligible influence on cell size and increases proliferation to 115%±10 when compared to control mock-depleted cells. Depletion was confirmed by transfecting cells with *Rictor-V5* 3 days after incubation with *Rictor* dsRNA. The number of cells expressing *Rictor-V5* was down by 90%. Cells were heat shocked analyzed after 5 days of incubation with ds RNA. *Sin1* depleted cells were slightly larger.

### Western blotting

Proteins were extracted using the following protocol. 10 larvae per condition were put in a test tube and snap frozen in liquid nitrogen. 100 µl of lysis buffer (1% Triton X-100, 50 mN Tris-HCl pH 7.5 and 150 mM NaCl, 10% glycerol, 50 mM NaF, 25 mM sodium glycero-phosphate, 1 mM sodium vanadate, 5 mM EDTA and protease inhibitor cocktail (from Roche, Rotkreuz, Switzerland) was added, and larvae were broken up with a small plastic pestle. 3×10^6^ S2 cells were extracted in 100 µl of lysis buffer. After centrifuging for 20 min at 14,000 rpm (20,800 ***g***) at 4°C, protein concentration was measured using BCA Protein assay (Pierce, Life Technologies, Zug, Switzerland) or Bradford. 50 μl of the supernatant (containing 100 μg protein from larvae, or 10–20 μg protein from S2 cells) was mixed with 11.2 µl of 5× SDS sample buffer and 1 µl DTT, boiled for 10 min, loaded on a SDS gel, and transferred onto nitrocellulose membrane. All blotting was performed in blocking buffer (TBST+5% milk powder). Phospho-S505 PKB/AKT (1:1000; Cell Signaling, BioConcept, Allschwil, Switzerland), total AKT (Cell Signaling, BioConcept, Allschwil, Switzerland), phospho-p38 (1:1000; Cell Signaling, BioConcept, Allschwil, Switzerland), active JNK (1:3000; Promega, Dübendorf, Switzerland), Erk1/2 (1:500; Sigma, Buchs, Switzerland) and Tubulin (1:10,000; Sigma, Buchs, Switzerland) antibodies were used. Secondary horseradish peroxidase (HRP)-conjugated anti-mouse-IgG and anti-rabbit-IgG (1:10,000) were from Amersham (GE Healthcare Life Sciences, Glattbrugg, Switzerland) and Jackson (Jackson ImmunoResearch Europe Ltd, Suffolk, UK), respectively.

### Immunofluorescence

S2 cells (grown at 26°C or incubated at 37°C) were fixed for 20 min at 4% PFA in PBS (except if otherwise indicated), processed for immunolabelling (Zacharogianni and Rabouille, 2013) and observed under a Leica SPE confocal microscope. Larval tissues were dissected from control or heat-stressed third-instar larvae, fixed with and processed for immunofluorescence as described previously (Zacharogianni and Rabouille, 2013). Larval tissues were dissected from larvae that were still alive after heat exposure and fixed with methanol. Hemocytes were recovered by ‘bleeding’ larvae for 5 min directly on a coverslip and fixing with PFA.

Primary antibodies used were mouse monoclonal anti-FMR1 (supernatant, diluted 1:10 for immunofluorescence) from DSHB (clone 5A11), rat anti-eIF4E (diluted 1:200 for immunofluorescence, with methanol fixation; Nakamura et al., 2004), rabbit polyclonal anti-Tral (diluted 1:200 for immunofluorescence; Wilhelm et al., 2005); rabbit polyclonal anti-cleaved *Drosophila* Dcp-1 (Asp216) (Cell signaling 9578, 1:100 for immunofluorescence). Secondary antibodies for immunofluorescence were goat anti-rabbit-IgG conjugated to Alexa Fluor 488, 568 and 647 (Life Technologies).

### Quantification of stress granule formation

The percentages of cells with stress granules were analysed from five independent experiments. Three to eight fields were analysed, comprising at least 150 cells per sample. The data were plotted in Excel and standard deviations and *P*-values were calculated with the same program.

For the maximum intensity plot (Fig. 5C), we measured the maximum intensity of FMR1 per cell on 150 mock-, *Rictor*- and *Sin1*-depleted S2 cells after exposure at 37°C for 1 h using ImageJ and plotted the data using Prism. The notion is that when stress granules form, the maximum intensity increases.

### qRT-PCR

Total RNA was isolated from larvae using Trizol Reagent (Invitrogen, Life Technologies, Zug, Switzerland). DNA contaminations were eliminated using Turbo DNA-free (Ambion, Life Technologies, Zug, Switzerland). cDNA was synthesized with SuperScript III RT (Invitrogen, Life Technologies, Zug, Switzerland). qRT-PCR for *HSP* genes was performed using LightCycler 480 SYBR Green I Mastermix (Roche, Rotkreuz, Switzerland) with the following primers: *HSP23*, 5′-TACTTGGCCCTGGTTGGAC-3′ and 5′-GCCCACCTGTTTCTCCAG-3′; *HSP26*, 5′-CTGCTTTCGCTTGTGGATGA-3′ and 5′-CAATCCCAGTCCAAGCTCGTA-3′; *HSP27*, 5′-TCCATGCCCACGATCTGTT-3′ and 5′-CTCCTCTCGTACGGCGAATAA-3′; *HSP83*, 5′-CGCGCATGAAGGATAACCA-3′ and 5′-TCCACGAAGGCAGAGTTGCT-3′; *HSP70AA*, 5′-CTATCCGGTGGCTGGACA-3′ and 5′-GCTCCTCCAGCTTGTGGTC-3′; *HSP70Ab*, 5′-CTATCCGGTGGCTGGACA-3′ and 5′-GCTCCTCCAGCTTGTGGTC-3′; *HSP70Ba:* 5′-GAAGGAGGAGTTCGACCACA-3′ and 5′-TGGTCATGATAGGGGAGCA-3′ *HSP70Bb*, 5′-CAACCAAGTAAATCAACTGCAACT-3′ and 5′-CGGTAACTTGTTGAAAGTATTCAGAG-3′; and *HSP70Bc*, 5′-CAAGTAAATCAACTGCAACTACTGAA-3′ and 5′-TGAAAGTATTCAGAGTTCTCTTCTGG-3′.

The expression ratio was calculated as a comparison of expression levels upon heat (36°C) versus expression levels under normal conditions (25°C) using REST-RG software (Pfaffl, 2001).

### Measuring translation inhibition efficiency by [^35^S]methionine and [^35^S]cysteine incorporation

S2 cells that were depleted for 5 days with mock, *Rictor* or *Sin1* siRNA were incubated at 26°C and 37°C for 1 h in 1 ml methionine- and cysteine-free SF-900 II medium (Life Technologies) supplemented with 1:1000 Schneider’s medium (Sigma) to bring the methionine and cysteine concentrations to 1 and 0.5 µM, respectively. Of note, incubation with this medium did not impair stress granule formation (not shown). After either 55 min or 105 min, 110 µCi (4.1 MBq) [^35^S]methionine and [^35^S]cysteine (easyTag™ Express protein labelling mix, Perkin Elmer) was added for 15 min. Incorporation was stopped by aspirating the medium and adding ice-cold HBSS (Invitrogen-BRL) containing 2 mM N-ethylmaleimide (Sigma) to block free sulfydryl groups (Li et al., 2015). Cells were lysed with 0.5% (v/v) Triton X-100 in ice cold MNT (20 mM MES, 100 mM NaCl, 30 mM Tris-HCl pH 7.5, containing 2 mM NEM and protease inhibitors cocktail (10 µg/ml each of chymostatin, leupeptin, antipain and pepstatin, 1 mM PMSF and 1 mM EDTA) buffer. Cell lysates were spun for 10 min at 15,000 ***g*** at 4°C to remove nuclei. After mixing (1:1) with SDS sample buffer [400 mM Tris-HCl pH 6.8, containing 6% (v/v) SDS, 20% (v/v) glycerol, 20 mM DTT and 0.01% (w/v) Bromophenol Blue], 10 µl were analysed using 10% SDS-PAGE gels that were stained by Coomassie Brilliant Blue R250, destained and dried. Radioactive signals were detected on Biomax MR films (Carestream Health, Rochester, NY).

The plots shown in Fig. 7C were obtained by using the gel function of ImageJ. Quantifications were performed with ImageQuant TL7.0 software (GE Healthcare) applying the provided minimal background subtraction on six autoradiograms of biological duplicates. To estimate translation efficiency, we subtracted the signals given by the two strong bands in the 37°C samples and normalized the reading to the intensity of the Coomassie staining (Fig. 7C).

### Cell survival and fitness upon and after heat stress

3×10^6^ S2 cells per experimental condition were plated in 3-cm plastic dishes (containing glass coverslips) and pretreated with 5 ml of Schneider's alone or supplemented with cycloheximide and puromycin for 30 min. The cells were then heat shocked for 3 h at 37°C. The cells are then washed twice with Schneider's (without drugs) and subsequently incubated for 16 h at 26°C. Cell viability was determined by exclusion of Trypan Blue. For each time point, 0.2 ml of cell suspension was mixed with 0.5 ml of 0.4% Trypan Blue for 6 min to evaluate the number of living cells that were counted using a hemocytometer. In parallel, cells were fixed and labelled for FMR1 and Dcp1 (see Immunofluorescence section). Experiments were repeated in triplicates.

## Supplementary Material

Supplementary Material

## References

[JCS168724C1] Anderson P. and Kedersha N. (2008). Stress granules: the Tao of RNA triage. *Trends Biochem. Sci.* 33, 141-150. 10.1016/j.tibs.2007.12.003 18291657

[JCS168724C2] Andjelkovic M., Jones P. F., Grossniklaus U., Cron P., Schier A. F., Dick M., Bilbe G. and Hemmings B. A. (1995). Developmental regulation of expression and activity of multiple forms of the Drosophila RAC protein kinase. *J. Biol. Chem.* 270, 4066-4075. 10.1074/jbc.270.8.4066 7876156

[JCS168724C3] Arimoto K., Fukuda H., Imajoh-Ohmi S., Saito H. and Takekawa M. (2008). Formation of stress granules inhibits apoptosis by suppressing stress-responsive MAPK pathways. *Nat. Cell Biol.* 10, 1324-1332. 10.1038/ncb1791 18836437

[JCS168724C4] Bang O.-S., Ha B.-G., Park E. K. and Kang S.-S. (2000). Activation of Akt is induced by heat shock and involved in suppression of heat-shock-induced apoptosis of NIH3T3 cells. *Biochem. Biophys. Res. Commun.* 278, 306-311. 10.1006/bbrc.2000.3805 11097835

[JCS168724C5] Beauchamp E. M. and Platanias L. C. (2013). The evolution of the TOR pathway and its role in cancer. *Oncogene* 32, 3923-3932. 10.1038/onc.2012.567 23246968

[JCS168724C6] Betz C. and Hall M. N. (2013). Where is mTOR and what is it doing there? *J. Cell Biol.* 203, 563-574. 10.1083/jcb.201306041 24385483 PMC3840941

[JCS168724C7] Cornu M., Oppliger W., Albert V., Robitaille A. M., Trapani F., Quagliata L., Fuhrer T., Sauer U., Terracciano L. and Hall M. N. (2014). Hepatic mTORC1 controls locomotor activity, body temperature, and lipid metabolism through FGF21. *Proc. Natl. Acad. Sci. USA* 111, 11592-11599. 10.1073/pnas.1412047111 25082895 PMC4136616

[JCS168724C8] Dibble C. C. and Manning B. D. (2013). Signal integration by mTORC1 coordinates nutrient input with biosynthetic output. *Nat. Cell Biol.* 15, 555-564. 10.1038/ncb2763 23728461 PMC3743096

[JCS168724C9] Eisinger-Mathason T. S. K., Andrade J., Groehler A. L., Clark D. E., Muratore-Schroeder T. L., Pasic L., Smith J. A., Shabanowitz J., Hunt D. F., Macara I. G. et al. (2008). Codependent functions of RSK2 and the apoptosis-promoting factor TIA-1 in stress granule assembly and cell survival. *Mol. Cell* 31, 722-736. 10.1016/j.molcel.2008.06.025 18775331 PMC2654589

[JCS168724C10] Eulalio A., Behm-Ansmant I., Schweizer D. and Izaurralde E. (2007). P-body formation is a consequence, not the cause, of RNA-mediated gene silencing. *Mol. Cell. Biol.* 27, 3970-3981. 10.1128/MCB.00128-07 17403906 PMC1900022

[JCS168724C11] Farny N. G., Kedersha N. L. and Silver P. A. (2009). Metazoan stress granule assembly is mediated by P-eIF2alpha-dependent and -independent mechanisms. *RNA* 15, 1814-1821. 10.1261/rna.1684009 19661161 PMC2743051

[JCS168724C12] Fournier M.-J., Coudert L., Mellaoui S., Adjibade P., Gareau C., Cote M.-F., Sonenberg N., Gaudreault R. C. and Mazroui R. (2013). Inactivation of the mTORC1-eukaryotic translation initiation factor 4E pathway alters stress granule formation. *Mol. Cell. Biol.* 33, 2285-2301. 10.1128/MCB.01517-12 23547259 PMC3648080

[JCS168724C13] Frias M. A., Thoreen C. C., Jaffe J. D., Schroder W., Sculley T., Carr S. A. and Sabatini D. M. (2006). mSin1 is necessary for Akt/PKB phosphorylation, and its isoforms define three distinct mTORC2s. *Curr. Biol.* 16, 1865-1870. 10.1016/j.cub.2006.08.001 16919458

[JCS168724C14] Fujimura K., Suzuki T., Yasuda Y., Murata M., Katahira J. and Yoneda Y. (2010). Identification of importin alpha1 as a novel constituent of RNA stress granules. *Biochim. Biophys. Acta* 1803, 865-871. 10.1016/j.bbamcr.2010.03.020 20362631

[JCS168724C15] Fujimura K., Sasaki A. T. and Anderson P. (2012). Selenite targets eIF4E-binding protein-1 to inhibit translation initiation and induce the assembly of non-canonical stress granules. *Nucleic Acids Res.* 40, 8099-8110. 10.1093/nar/gks566 22718973 PMC3439927

[JCS168724C16] Gareau C., Houssin E., Martel D., Coudert L., Mellaoui S., Huot M.-E., Laprise P. and Mazroui R. (2013). Characterization of fragile X mental retardation protein recruitment and dynamics in Drosophila stress granules. *PLoS ONE* 8, e55342 10.1371/journal.pone.0055342 23408971 PMC3567066

[JCS168724C17] Glatter T., Schittenhelm R. B., Rinner O., Roguska K., Wepf A., Junger M. A., Kohler K., Jevtov I., Choi H., Schmidt A. et al. (2011). Modularity and hormone sensitivity of the Drosophila melanogaster insulin receptor/target of rapamycin interaction proteome. *Mol. Syst. Biol.* 7, 547 10.1038/msb.2011.79 22068330 PMC3261712

[JCS168724C18] Heitman J., Movva N. R. and Hall M. N. (1991). Targets for cell cycle arrest by the immunosuppressant rapamycin in yeast. *Science* 253, 905-909. 10.1126/science.1715094 1715094

[JCS168724C19] Hietakangas V. and Cohen S. M. (2007). Re-evaluating AKT regulation: role of TOR complex 2 in tissue growth. *Genes Dev.* 21, 632-637. 10.1101/gad.416307 17369395 PMC1820936

[JCS168724C20] Ho H.-L., Shiau Y.-S. and Chen M.-Y. (2005). Saccharomyces cerevisiaeTSC11/AVO3 participates in regulating cell integrity and functionally interacts with components of the Tor2 complex. *Curr. Genet.* 47, 273-288. 10.1007/s00294-005-0570-8 15809876

[JCS168724C21] Ikeda K., Morigasaki S., Tatebe H., Tamanoi F. and Shiozaki K. (2008). Fission yeast TOR complex 2 activates the AGC-family Gad8 kinase essential for stress resistance and cell cycle control. *Cell Cycle* 7, 358-364. 10.4161/cc.7.3.5245 18235227 PMC2274895

[JCS168724C22] Ikenoue T., Inoki K., Yang Q., Zhou X. and Guan K.-L. (2008). Essential function of TORC2 in PKC and Akt turn motif phosphorylation, maturation and signalling. *EMBO J.* 27, 1919-1931. 10.1038/emboj.2008.119 18566587 PMC2486275

[JCS168724C23] Jacinto E., Loewith R., Schmidt A., Lin S., Rüegg M. A., Hall A. and Hall M. N. (2004). Mammalian TOR complex 2 controls the actin cytoskeleton and is rapamycin insensitive. *Nat. Cell Biol.* 6, 1122-1128. 10.1038/ncb1183 15467718

[JCS168724C24] Jacinto E., Facchinetti V., Liu D., Soto N., Wei S., Jung S. Y., Huang Q., Qin J. and Su B. (2006). SIN1/MIP1 maintains rictor-mTOR complex integrity and regulates Akt phosphorylation and substrate specificity. *Cell* 127, 125-137. 10.1016/j.cell.2006.08.033 16962653

[JCS168724C25] Johnson G. L. and Lapadat R. (2002). Mitogen-activated protein kinase pathways mediated by ERK, JNK, and p38 protein kinases. *Science* 298, 1911-1912. 10.1126/science.1072682 12471242

[JCS168724C26] Jünger M. A., Rintelen F., Stocker H., Wasserman J. D., Végh M., Radimerski T., Greenberg M. E. and Hafen E. (2003). The Drosophila forkhead transcription factor FOXO mediates the reduction in cell number associated with reduced insulin signaling. *J. Biol.* 2, 20 10.1186/1475-4924-2-20 12908874 PMC333403

[JCS168724C27] Kedersha N. L., Gupta M., Li W., Miller I. and Anderson P. (1999). RNA-binding proteins TIA-1 and TIAR link the phosphorylation of eIF-2alpha to the assembly of mammalian stress granules. *J. Cell Biol.* 147, 1431-1442. 10.1083/jcb.147.7.1431 10613902 PMC2174242

[JCS168724C28] Kim D.-H., Sarbassov D. D., Ali S. M., King J. E., Latek R. R., Erdjument-Bromage H., Tempst P. and Sabatini D. M. (2002). mTOR interacts with raptor to form a nutrient-sensitive complex that signals to the cell growth machinery. *Cell* 110, 163-175. 10.1016/S0092-8674(02)00808-5 12150925

[JCS168724C29] Kim W. J., Back S. H., Kim V., Ryu I. and Jang S. K. (2005). Sequestration of TRAF2 into stress granules interrupts tumor necrosis factor signaling under stress conditions. *Mol. Cell. Biol.* 25, 2450-2462. 10.1128/MCB.25.6.2450-2462.2005 15743837 PMC1061607

[JCS168724C30] Kondylis V. and Rabouille C. (2003). A novel role for dp115 in the organization of tER sites in Drosophila. *J. Cell Biol.* 162, 185-198. 10.1083/jcb.200301136 12876273 PMC2172793

[JCS168724C31] Kondylis V., van Nispen tot Pannerden H. E., Herpers B., Friggi-Grelin F. and Rabouille C. (2007). The golgi comprises a paired stack that is separated at G2 by modulation of the actin cytoskeleton through Abi and Scar/WAVE. *Dev. Cell* 12, 901-915. 10.1016/j.devcel.2007.03.008 17543863

[JCS168724C32] Kwon S., Zhang Y. and Matthias P. (2007). The deacetylase HDAC6 is a novel critical component of stress granules involved in the stress response. *Genes Dev.* 21, 3381-3394. 10.1101/gad.461107 18079183 PMC2113037

[JCS168724C33] Laplante M. and Sabatini D. M. (2012). mTOR signaling in growth control and disease. *Cell* 149, 274-293. 10.1016/j.cell.2012.03.017 22500797 PMC3331679

[JCS168724C34] Li S., Brown M. S. and Goldstein J. L. (2010). Bifurcation of insulin signaling pathway in rat liver: mTORC1 required for stimulation of lipogenesis, but not inhibition of gluconeogenesis. *Proc. Natl. Acad. Sci. USA* 107, 3441-3446. 10.1073/pnas.0914798107 20133650 PMC2840492

[JCS168724C35] Li X., van Oers M. M., Vlak J. M. and Braakman I. (2015). Folding of influenza virus hemagglutinin in insect cells is fast and efficient. *J. Biotechnol.* 203, 77-83. 10.1016/j.jbiotec.2015.03.018 25828453

[JCS168724C36] Lindquist S. (1986). The heat-shock response. *Annu. Rev. Biochem.* 55, 1151-1191. 10.1146/annurev.bi.55.070186.005443 2427013

[JCS168724C37] Loewith R. and Hall M. N. (2011). Target of rapamycin (TOR) in nutrient signaling and growth control. *Genetics* 189, 1177-1201. 10.1534/genetics.111.133363 22174183 PMC3241408

[JCS168724C38] Loewith R., Jacinto E., Wullschleger S., Lorberg A., Crespo J. L., Bonenfant D., Oppliger W., Jenoe P. and Hall M. N. (2002). Two TOR complexes, only one of which is rapamycin sensitive, have distinct roles in cell growth control. *Mol. Cell* 10, 457-468. 10.1016/S1097-2765(02)00636-6 12408816

[JCS168724C39] Manning B. D. and Cantley L. C. (2007). AKT/PKB signaling: navigating downstream. *Cell* 129, 1261-1274. 10.1016/j.cell.2007.06.009 17604717 PMC2756685

[JCS168724C40] Martin J., Masri J., Bernath A., Nishimura R. N. and Gera J. (2008). Hsp70 associates with Rictor and is required for mTORC2 formation and activity. *Biochem. Biophys. Res. Commun.* 372, 578-583. 10.1016/j.bbrc.2008.05.086 18505677 PMC2512964

[JCS168724C41] McEwen E., Kedersha N., Song B., Scheuner D., Gilks N., Han A., Chen J.-J., Anderson P. and Kaufman R. J. (2005). Heme-regulated inhibitor kinase-mediated phosphorylation of eukaryotic translation initiation factor 2 inhibits translation, induces stress granule formation, and mediates survival upon arsenite exposure. *J. Biol. Chem.* 280, 16925-16933. 10.1074/jbc.M412882200 15684421

[JCS168724C42] Nadeau S. I. and Landry J. (2007). Mechanisms of activation and regulation of the heat shock-sensitive signaling pathways. *Adv. Exp. Med. Biol.* 594, 100-113. 10.1007/978-0-387-39975-1_10 17205679

[JCS168724C43] Nakamura A., Sato K. and Hanyu-Nakamura K. (2004). Drosophila cup is an eIF4E binding protein that associates with Bruno and regulates oskar mRNA translation in oogenesis. *Dev. Cell* 6, 69-78. 10.1016/S1534-5807(03)00400-3 14723848

[JCS168724C44] Oh W. J. and Jacinto E. (2011). mTOR complex 2 signaling and functions. *Cell Cycle* 10, 2305-2316. 10.4161/cc.10.14.16586 21670596 PMC3322468

[JCS168724C45] Oh W. J., Wu C.-c., Kim S. J., Facchinetti V., Julien L.-A., Finlan M., Roux P. P., Su B. and Jacinto E. (2010). mTORC2 can associate with ribosomes to promote cotranslational phosphorylation and stability of nascent Akt polypeptide. *EMBO J.* 29, 3939-3951. 10.1038/emboj.2010.271 21045808 PMC3020639

[JCS168724C46] Ohn T., Kedersha N., Hickman T., Tisdale S. and Anderson P. (2008). A functional RNAi screen links O-GlcNAc modification of ribosomal proteins to stress granule and processing body assembly. *Nat. Cell Biol.* 10, 1224-1231. 10.1038/ncb1783 18794846 PMC4318256

[JCS168724C47] Pfaffl M. W. (2001). A new mathematical model for relative quantification in real-time RT-PCR. *Nucleic Acids Res.* 29, e45 10.1093/nar/29.9.e45 11328886 PMC55695

[JCS168724C48] Reiling J. H. and Sabatini D. M. (2006). Stress and mTORture signaling. *Oncogene* 25, 6373-6383. 10.1038/sj.onc.1209889 17041623

[JCS168724C49] Sabatini D. M. (2006). mTOR and cancer: insights into a complex relationship. *Nat. Rev. Cancer* 6, 729-734. 10.1038/nrc1974 16915295

[JCS168724C50] Sabatini D. M., Erdjument-Bromage H., Lui M., Tempst P. and Snyder S. H. (1994). RAFT1: a mammalian protein that binds to FKBP12 in a rapamycin-dependent fashion and is homologous to yeast TORs. *Cell* 78, 35-43. 10.1016/0092-8674(94)90570-3 7518356

[JCS168724C51] Sama R. R. K., Ward C. L., Kaushansky L. J., Lemay N., Ishigaki S., Urano F. and Bosco D. A. (2013). FUS/TLS assembles into stress granules and is a prosurvival factor during hyperosmolar stress. *J. Cell Physiol.* 228, 2222-2231. 10.1002/jcp.24395 23625794 PMC4000275

[JCS168724C52] Sancak Y., Bar-Peled L., Zoncu R., Markhard A. L., Nada S. and Sabatini D. M. (2010). Ragulator-Rag complex targets mTORC1 to the lysosomal surface and is necessary for its activation by amino acids. *Cell* 141, 290-303. 10.1016/j.cell.2010.02.024 20381137 PMC3024592

[JCS168724C53] Sarbassov D. D., Ali S. M., Kim D.-H., Guertin D. A., Latek R. R., Erdjument-Bromage H., Tempst P. and Sabatini D. M. (2004). Rictor, a novel binding partner of mTOR, defines a rapamycin-insensitive and raptor-independent pathway that regulates the cytoskeleton. *Curr. Biol.* 14, 1296-1302. 10.1016/j.cub.2004.06.054 15268862

[JCS168724C54] Sarbassov D. D., Guertin D. A., Ali S. M. and Sabatini D. M. (2005). Phosphorylation and regulation of Akt/PKB by the rictor-mTOR complex. *Science* 307, 1098-1101. 10.1126/science.1106148 15718470

[JCS168724C55] Scheid M. P. and Woodgett J. R. (2001). PKB/AKT: functional insights from genetic models. *Nat. Rev. Mol. Cell Biol.* 2, 760-768. 10.1038/35096067 11584303

[JCS168724C56] Schmidt A., Kunz J. and Hall M. N. (1996). TOR2 is required for organization of the actin cytoskeleton in yeast. *Proc. Natl. Acad. Sci. USA* 93, 13780-13785. 10.1073/pnas.93.24.13780 8943012 PMC19424

[JCS168724C57] Schonbrun M., Laor D., Lopez-Maury L., Bahler J., Kupiec M. and Weisman R. (2009). TOR complex 2 controls gene silencing, telomere length maintenance, and survival under DNA-damaging conditions. *Mol. Cell. Biol.* 29, 4584-4594. 10.1128/MCB.01879-08 19546237 PMC2725747

[JCS168724C58] Sengupta S., Peterson T. R., Laplante M., Oh S. and Sabatini D. M. (2010). mTORC1 controls fasting-induced ketogenesis and its modulation by ageing. *Nature* 468, 1100-1104. 10.1038/nature09584 21179166

[JCS168724C59] Shaw M., Cohen P. and Alessi D. R. (1998). The activation of protein kinase B by H2O2 or heat shock is mediated by phosphoinositide 3-kinase and not by mitogen-activated protein kinase-activated protein kinase-2. *Biochem. J.* 336, 241-246. 9806907 10.1042/bj3360241 PMC1219864

[JCS168724C60] Shiota C., Woo J.-T., Lindner J., Shelton K. D. and Magnuson M. A. (2006). Multiallelic disruption of the rictor gene in mice reveals that mTOR complex 2 is essential for fetal growth and viability. *Dev. Cell* 11, 583-589. 10.1016/j.devcel.2006.08.013 16962829

[JCS168724C61] Soukas A. A., Kane E. A., Carr C. E., Melo J. A. and Ruvkun G. (2009). Rictor/TORC2 regulates fat metabolism, feeding, growth, and life span in Caenorhabditis elegans. *Genes Dev.* 23, 496-511. 10.1101/gad.1775409 19240135 PMC2648650

[JCS168724C62] Soulard A., Cohen A. and Hall M. N. (2009). TOR signaling in invertebrates. *Curr. Opin. Cell Biol.* 21, 825-836. 10.1016/j.ceb.2009.08.007 19767189

[JCS168724C63] Takahara T. and Maeda T. (2012). Transient sequestration of TORC1 into stress granules during heat stress. *Mol. Cell* 47, 242-252. 10.1016/j.molcel.2012.05.019 22727621

[JCS168724C64] Takai H., Xie Y., de Lange T. and Pavletich N. P. (2010). Tel2 structure and function in the Hsp90-dependent maturation of mTOR and ATR complexes. *Genes Dev.* 24, 2019-2030. 10.1101/gad.1956410 20801936 PMC2939364

[JCS168724C65] Thedieck K., Holzwarth B., Prentzell M. T., Boehlke C., Kläsener K., Ruf S., Sonntag A. G., Maerz L., Grellscheid S.-N., Kremmer E. et al. (2013). Inhibition of mTORC1 by astrin and stress granules prevents apoptosis in cancer cells. *Cell* 154, 859-874. 10.1016/j.cell.2013.07.031 23953116

[JCS168724C66] Urban J., Soulard A., Huber A., Lippman S., Mukhopadhyay D., Deloche O., Wanke V., Anrather D., Ammerer G., Riezman H. et al. (2007). Sch9 is a major target of TORC1 in Saccharomyces cerevisiae. *Mol. Cell* 26, 663-674. 10.1016/j.molcel.2007.04.020 17560372

[JCS168724C67] van der Laan A. M. A., Vangemert A. M. C., Dirks R. W., Noordermeer J. N., Fradkin L. G., Tanke H. J. and Jost C. R. (2012). mRNA cycles through hypoxia-induced stress granules in live Drosophila embryonic muscles. *Int. J. Dev. Biol.* 56, 701-709. 10.1387/ijdb.103172al 23319346

[JCS168724C68] Wang T., Blumhagen R., Lao U., Kuo Y. and Edgar B. A. (2012). LST8 regulates cell growth via target-of-rapamycin complex 2 (TORC2). *Mol. Cell. Biol.* 32, 2203-2213. 10.1128/MCB.06474-11 22493059 PMC3372270

[JCS168724C69] Weisman R. and Choder M. (2001). The fission yeast TOR homolog, tor1+, is required for the response to starvation and other stresses via a conserved serine. *J. Biol. Chem.* 276, 7027-7032. 10.1074/jbc.M010446200 11096119

[JCS168724C70] Wen W.-L., Stevenson A. L., Wang C.-Y., Chen H.-J., Kearsey S. E., Norbury C. J., Watt S., Bahler J. and Wang S.-W. (2010). Vgl1, a multi-KH domain protein, is a novel component of the fission yeast stress granules required for cell survival under thermal stress. *Nucleic Acids Res.* 38, 6555-6566. 10.1093/nar/gkq555 20547592 PMC2965253

[JCS168724C71] Wilhelm J. E., Buszczak M. and Sayles S. (2005). Efficient protein trafficking requires trailer hitch, a component of a ribonucleoprotein complex localized to the ER in Drosophila. *Dev. Cell* 9, 675-685. 10.1016/j.devcel.2005.09.015 16256742

[JCS168724C72] Wilkinson M. G., Pino T. S., Tournier S., Buck V., Martin H., Christiansen J., Wilkinson D. G. and Millar J. B. A. (1999). Sin1: an evolutionarily conserved component of the eukaryotic SAPK pathway. *EMBO J.* 18, 4210-4221. 10.1093/emboj/18.15.4210 10428959 PMC1171497

[JCS168724C73] Wippich F., Bodenmiller B., Trajkovska M. G., Wanka S., Aebersold R. and Pelkmans L. (2013). Dual specificity kinase DYRK3 couples stress granule condensation/dissolution to mTORC1 signaling. *Cell* 152, 791-805. 10.1016/j.cell.2013.01.033 23415227

[JCS168724C74] Yamasaki S., Stoecklin G., Kedersha N., Simarro M. and Anderson P. (2007). T-cell intracellular antigen-1 (TIA-1)-induced translational silencing promotes the decay of selected mRNAs. *J. Biol. Chem.* 282, 30070-30077. 10.1074/jbc.M706273200 17711853

[JCS168724C75] Yang Q., Inoki K., Ikenoue T. and Guan K.-L. (2006). Identification of Sin1 as an essential TORC2 component required for complex formation and kinase activity. *Genes Dev.* 20, 2820-2832. 10.1101/gad.1461206 17043309 PMC1619946

[JCS168724C76] Zacharogianni M. and Rabouille C. (2013). Trafficking along the secretory pathway in Drosophila cell line and tissues: a light and electron microscopy approach. *Methods Cell Biol.* 118, 35-49. 10.1016/b978-0-12-417164-0.00003-3 24295299

[JCS168724C77] Zinzalla V., Stracka D., Oppliger W. and Hall M. N. (2011). Activation of mTORC2 by association with the ribosome. *Cell* 144, 757-768. 10.1016/j.cell.2011.02.014 21376236

[JCS168724C78] Zoncu R., Efeyan A. and Sabatini D. M. (2011). mTOR: from growth signal integration to cancer, diabetes and ageing. *Nat. Rev. Mol. Cell Biol.* 12, 21-35. 10.1038/nrm3025 21157483 PMC3390257

