# Supplementary material for: TORC2 mediates the heat stress response in Drosophila by promoting the formation of stress granules
Source: J Cell Sci. 2015 Jul 15;128(14):2497–508. doi: 10.1242/jcs.168724 (PMC4510851; doi:10.1242/jcs.168724)
Supplement: Supplementary Material [file supp_128_14_2497__index.html]

Supplementary Material 

# TORC2 mediates the heat stress response in *Drosophila* by promoting the formation of stress granules

## JCS168724 Supplementary Material

- Supplementary Material
