# Supplementary material for: TORC2 mediates the heat stress response in Drosophila by promoting the formation of stress granules
Source: J Cell Sci. 2015 Jul 15;128(14):2497–508. doi: 10.1242/jcs.168724 (PMC4510851; doi:10.1242/jcs.168724)
Supplement: Supplementary Material [file supp_jcs.168724_JCS168724supp.pdf]

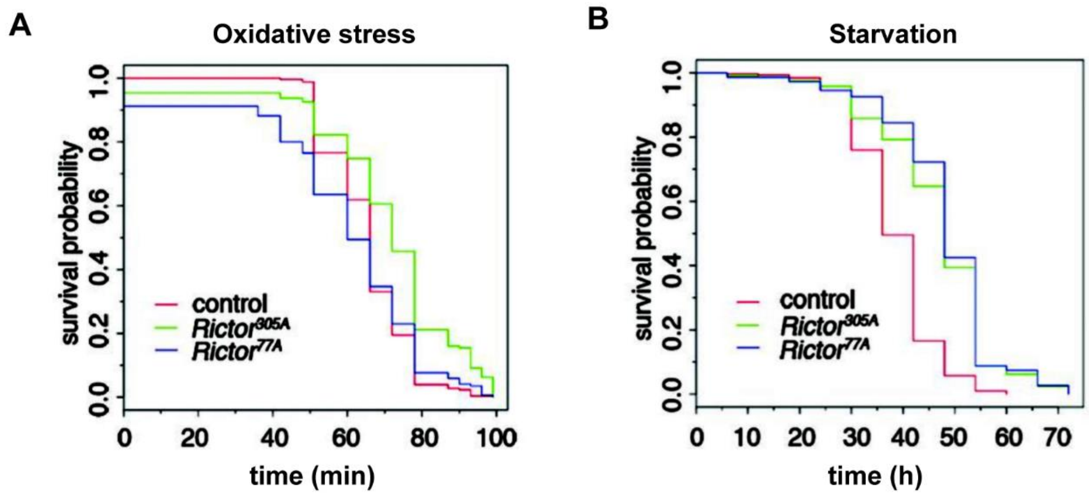

Suppl. Figure S1: TORC2 mutant flies are not sensitive to oxidative stress or starvation.
A: Quantitation of the sensitivity to oxidative stress (5% H2O2) of control flies (1A, n= 257), Rictor77A (n=170) and Rictor305A (n=175) expressed as the probability of surviving.
p for Rictor77A and 305A vs. control is not statistically significant.
B: Quantitation of the sensitivity to complete starvation of control flies (1A, n=313), Rictor77A (n=148), Rictor305A (n=241) expressed as probability of surviving.
p-value for Rictor vs. control is $p<0.001$.

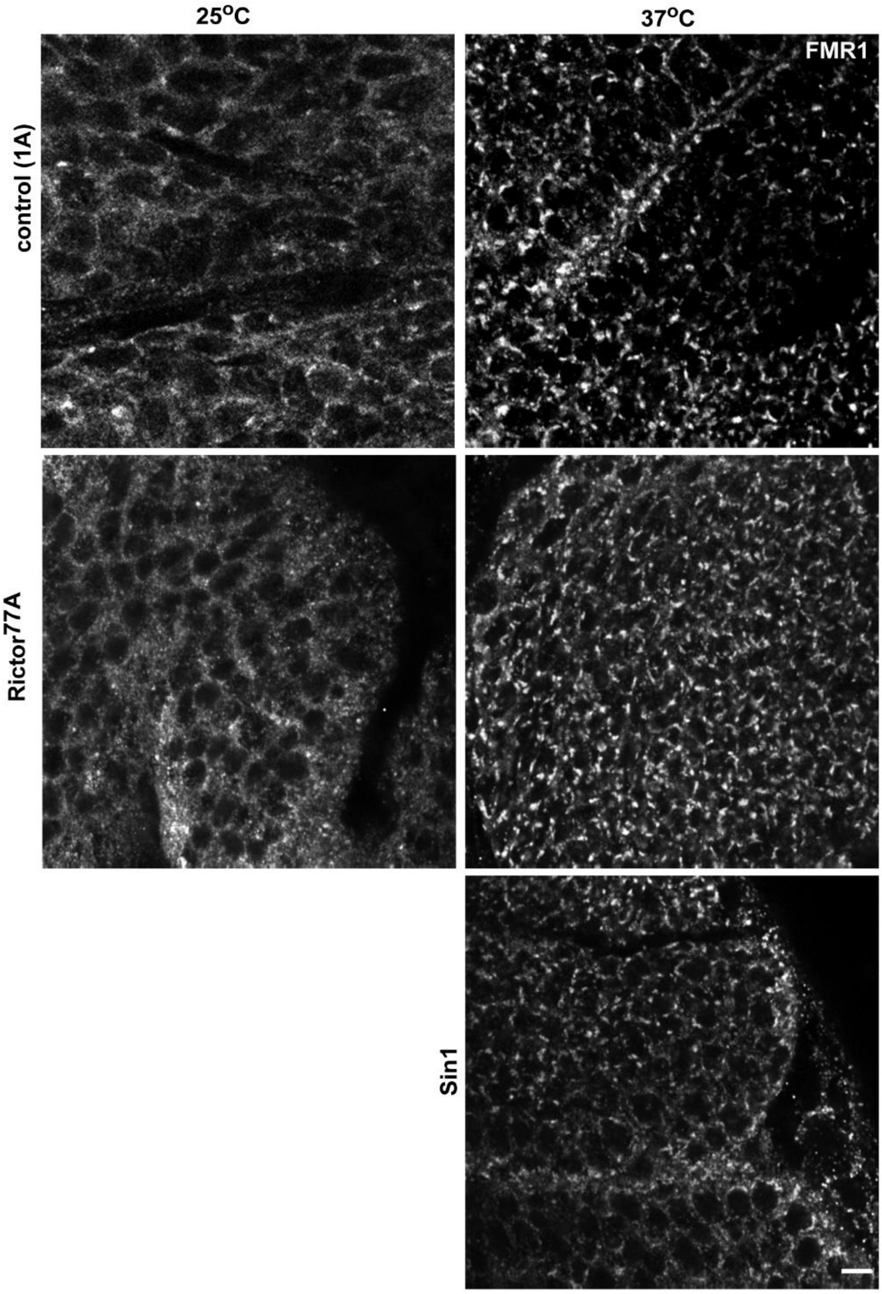

Suppl. Figure S2: Stress granule formation in larval brain does not appear to depend on TORC2.
Immunofluorescence visualisation of FMR1 in larval brains after 2h at 26 and 37°C. Note that a pattern consistent with stress granule formation forms in heat stressed brains, but in control (1A), Rictor and Sin1 mutant to the same extent.
Scale bar: 50um
